# Assessment of Safe Motherhood Health Service Coverage, Birth Defects Detection and Child Disability Prevention Using Lot Quality Assurance Sampling in Central Uganda

**DOI:** 10.24248/eahrj.v7i1.703

**Published:** 2023-07-12

**Authors:** Edith Akankwasa, Willy Kamya, Moses Sendijja, Janet Mudoola, Mathias Lwenge, Robert Anguyo DDM Onzima, Daniel Kasozi, Peter Byansi, Simon Peter Katongole

**Affiliations:** a Mildmay Institute of Health Sciences, Kampala, Uganda; b Department of International Public Health, Monitoring, Evaluation, Technical Assistance and Research (METRe) Group, Liverpool School of Tropical Medicine (LSTM), Liverpool, United Kingdom; c The United States Agency for International Development, US Embassy, Kampala Uganda; d School of Public Health, Gudie University Project, Kampala, Uganda

## Abstract

**Introduction:**

It is crucial to have satisfactory coverage of safe motherhood services in order to prevent birth defects and child disabilities. Mildmay Uganda Institute of Health Sciences (MIHS) implemented a safe motherhood project aimed at preventing birth defects and child disabilities.

**Methods:**

Three years after the project's implementation, a rapid cross-sectional health facility survey was conducted in 4 districts of central Uganda to assess the coverage of key safe motherhood and early childhood services. The Lot Quality Assurance Sampling approach was used to assess coverage of 16 indicators in the areas of ANC, skilled birth attendance, early childhood care, postnatal care, and knowledge about child disability prevention. A Decision Rule was set at 80% upper threshold to classify the performance of health facilities at the district level.

**Results:**

The survey found that there was variation in performance across indicators and districts. All districts achieved the 80% coverage target in ANC first visit, mothers who received at least two doses of Fansidar for intermittent preventive treatment of malaria in pregnancy, and mothers with knowledge of the action to take in case they suspect childhood disability. Folic acid supplementation during pregnancy and screening for birth defects using the Appearance, Pulse, Grimace, Activity, and Respiration (APGAR) score had overall coverage above the target, but one district each had coverage below target in each of these 2 indicators. The coverage target was not reached in the rest of the survey indicators in each of the districts.

**Conclusion:**

Well-performing districts, especially in indicators with inconsistent performance, offer valuable insights for learning and adapting interventions in districts that do not meet the desired coverage of those particular indicators. Considering the disparities in performance among different indicators and districts, project planners should adopt, modify and implement successful strategies in districts and indicators that perform well. By doing so, they can enhance the performance of under performing districts or indicators.

## BACKGROUND

For over 3 decades now, the World Health Organization (WHO) has been emphasising the importance of providing effective safe-motherhood care to ensure positive pregnancy outcomes.^1^ The components of safe motherhood including; timely attendance at antenatal care, skilled birth attendance, early detection and management of pregnancy complications and effective postnatal care for all mothers. All these are essential components of safe motherhood crucial in preventing birth defects and child disabilities.^2–4^ Maternal and child health services, including reproductive health are typically incorporated into primary healthcare services in most countries, thus making the prevention of birth defects an integral part of these services at all levels of primary healthcare.^3^

During antenatal care, mothers receive health education on what to do and what not to do to prevent birth defects. They also receive supplements such as Folic Acid, examinations and investigations for the presence of any infections and treatment.^5^ Mothers attending Antenatal Care (ANC) are also educated about the avoidance of behaviours and practices that could result in birth defects such as; use of alcohol, smoking, illicit drugs, drugs not prescribed by a healthcare professional and exposure to harmful substances.^3,6^ Supplements such as Folic Acid help prevent brain and spinal defects such as neural tube defects.^7,8^ Iron supplementation in the first and second trimesters of pregnancy can also help mothers maintain adequate iron levels, prevent the development of psychomotor challenges, and prevent the impairment of cognitive development.^7^ ANC care also includes screening for diseases such as syphilis and rubella that could contribute to birth defects, and treatment is provided where necessary.^9^

Although safe motherhood programs have numerous benefits, the correlation between safe motherhood and birth defects and disability prevention has often been overlooked. To address this issue, the Mildmay Institute of Health Sciences (MIHS) implemented the Child Disability Management and Rehabilitation (CDR) project in 4 districts in Central Uganda (Mityana, Mubende, Luwero, and Kassanda). The goal was to increase the coverage of safe motherhood services in order to prevent birth defects and child disability. The project worked through existing district health systems to strengthen the capacity of health facilities, health care providers and community resource persons to provide quality child disability prevention, detection and rehabilitation services. The project also trained village health teams and community resource persons to detect birth defects in children and link them to appropriate services.

Additionally, the project provided advocacy for child disability services, created and supported community-based rehabilitation services, and economically empowered families with children with disabilities to access maternal and child health services without financial hardship. The project was implemented in 61 health facilities and their catchment areas across the 4 districts.

As part of the project's learning process, the primary objective of this study was to assess the coverage of safe motherhood indicators at the end of the 3-year project period. The specific objective was to determine the extent to which health facilities in their respective catchment areas provided safe motherhood services to mothers and to record health workers' and caregivers' explanations about the status of coverage for specific safe motherhood and child disability prevention indicators.

By studying the effectiveness of this safe motherhood and child disability implementation project and interventions, it helped in identifying successful strategies and areas that required modification. This information is vital in enhancing future project implementation and improving health outcomes for mothers and children. Additionally, comprehending the obstacles to effective implementation can guide policy and program decisions, facilitating the resolution of systemic issues and the enhancement of district health systems. Ultimately, the aim is to offer mothers and children high-quality healthcare services that prevent birth defects and manage disabilities, resulting in improved health outcomes and better quality of life for all.

## MATERIALS AND METHODS

### Study Setting

The study was conducted in 4 districts; Mityana, Mubende, Luwero, and Kassanda where the Mildmay Institute of Health Sciences had implemented interventions for safe motherhood to prevent birth defects and child disability. The interventions started in Mubende district in July of 2014/2015 financial year and were expanded to Mityana in the 2015/2016 financial year. Luwero was added in the 2016/2017 financial year, and Kassanda was included in 2017/2018 after it was carved out of Mubende district.

The interventions ended in June 2021.

These interventions included among others, training midwives to improve their knowledge and skills in providing safe motherhood care, improving public health education about safe motherhood to mothers who present for ANC at the Primary Healthcare Centres (PHCs) as well as health education within the communities using community towers and radios. Health education also aimed at improving skilled birth attendance and increasing the number of mothers attending postnatal care with their children. The project also provided tools to the Healthcare Facilities aimed at increasing access to and provision of antenatal services during pregnancy and during childbirth. The use of the ultrasound scan was emphasised from 20 to 24 weeks of pregnancy in order to detect birth defects. In the event that birth defects were detected during pregnancy, the providers were supposed to refer such a mother for professional and specialised handling for the remaining period of pregnancy and during birth.

### Study Design

The study utilised the Rapid Health Facility Assessment (r-HFA) Lot Quality Assurance Survey (LQAS) approach. This methodology has been widely applied in various studies to evaluate the outcomes related to maternal and child health.^10,11^ This approach is utilised to evaluate the standard and extent of services provided in primary healthcare centres.^11^ This study used an evaluation approach to measure the coverage of safe motherhood services and early childhood services, which are important for preventing birth defects and child disabilities. The approach involved selecting a sample of health facilities and determining the extent to which they provided safe motherhood services to mothers in their catchment areas, based on established coverage standards using the binomial LQAS method. Additionally, qualitative interviews were conducted with midwives to gain insights into the factors that influenced the delivery of the services being assessed.

### Sampling Frame and Sample Size

The study involved a sampling frame of 61 health facilities; 24 in Mityana, 10 in Mubende, and 13 in each of Luwero and Kassanda. The health facilities were a mix of government and private not-for-profit organisations, including 12 Health Centre IIs, 38 Health Centre IIIs, 8 Health Centre IVs, 2 general hospitals, and 1 Regional Referral Hospital (RRH). Sample sizes of health facilities in each district were calculated using the Hypergeometric LQAS principles.^12^ These were used because of the finite population of health facilities in each of the district. The study estimated the sample size (n) and decision rule (DR) for each district using the algorithm for hypergeometric LQAS by following the steps below;

i. For a district with N population of health facilities, we started with n=1, DR=0.

ii. Calculated the rounded upper threshold number, H = round(pU*N), and the rounded lower threshold number, L = round(pL*N)

iii. The alpha (α), was calculated as the Cumulative Distribution Function (CDF) of the hypergeometric distribution, whereby; alpha=CDF(DR, N, H, n) and beta (β) of 1 – CDF(DR, N, L, n)

iv. If α<= α_threshold and β<= β_threshold, then the solution is (n, DR), otherwise we continued as in step (v)

v. Where DR=n then we increased n by 1 and set DR at 0. Otherwise increased DR by 1 and returned to step (ii)

In order to ensure an optimum sample size (n) for a given district where the estimated n ≤7, n was rounded upwards to 8 and DR estimated at n=8, all the other parameters described below kept constant.

The study divided the program area into 4 districts and each district formed a Supervision Area (SA). The SA was the smallest division used to assess the health facility's performance in delivering Safe Motherhood (SMH) services, and a Decision Rule (DR) was used to determine if the district was performing well or not. The DR is the cut-off point for the optimal performance of Healthcare Facilities (HFs) for each indicator for the calculated sample size (n) in each district. To identify poor performance, the hypergeometric LQAS sampling technique required an upper threshold (pU) and lower threshold (pL) set at 80% and 50%, respectively. The study allowed an acceptable α (α_threshold) and β (β_threshold) error rates of 0.1 each. The α error is the probability of misclassifying a district Supervision Area (SA) as having performed well when it has poor performance in reality.^13^ In this study, the term “β error” refers to the probability of wrongly classifying a district's performance as poor on a specific indicator, when in reality it is actually performing well.^11^ (Table 1)

**TABLE 1:** Sample Size of the Health Facilities (HFs) in the District

District	HC II	HC III	HC IV	Hospital	Eligible HFs (total)	n	Actual α error	Actual β error	DR
Mityana	5	15	3	1	24	13	0.0303	0.0498	9
Mubende	2	7	0	1	10	8	0	0.0303	6
Luwero	0	10	3	1	14	10	0	0.0350	7
Kassanda	5	6	2	0	13	10	0	0.0699	7
Total	12	38	8	3	61	41			

The total sample size for the study was 41 Healthcare Facilities (HFs); 13 in Mityana, 8 in Mubende, 10 in Luwero and 10 in Kassanda districts. The HFs in each district were sampled using simple random sampling without replacement.

To evaluate the performance of each healthcare facility (HF) regarding a particular indicator, the researchers used the binomial LQAS model and selected 6 data points from each HF or its catchment area. This resulted in a sample size of 246 data points for the 41 HFs. If at least 5 out of the 6 sampled data points had the characteristic of interest, the HF was classified as having satisfactory performance for the indicator. This method of classification has been used in other studies previously.^10^ The DR of 5 was obtained using a sample size of 6 with pU=95% and pL=50%, as well as α_threshold and β_threshold of 0.11 each. The actual α and β errors varied from one district to another due to differences in HF population across districts.

In-depth interviews were conducted with 12 midwives and 14 mothers of children aged 0 to 11 months as part of the qualitative data collection process to provide insights into the quantitative findings presented in the data collection section.

### Sampling of Interview Locations (Villages) in the HF Catchment Areas of Sampled HFs

To determine the coverage of other SMH indicators crucial to preventing and detecting birth defects at birth, a community-based survey was conducted in each sampled HF's catchment area, in addition to the HF-based assessment of some indicators. A list of all the villages in the catchment area of the HF was obtained from the HF's in-charge and weighted for population size using a 3-category weighting scale (3 for high population, 2 for medium population, and 1 for low population). The weighting of the villages was agreed upon by consensus between the health workers and HF in-charge to ensure reliability. Six interview locations were then selected from each HF's list of weighted villages using simple random sampling with replacement, resulting in a sample size of 246 mothers of children aged 0 to 11 months for the community survey. The selected interview location/village was then segmented with the help of a village guide, and 1 segment was randomly sampled. This process was repeated until a segment containing less than 15 households was reached, based on previous studies, to make it easier to identify the starting point.^14,15^

The final segment was used to list all the households, using the names of the heads of households or other identifiers. From this list, one household was selected at random as the reference household, and the research assistants began the search for an eligible respondent from the next-nearest household starting from the front door of the reference household. Once data collection was completed in one village/interview location, the research assistants moved on to the next sampled village. Additionally, 6 children were randomly selected from the maternity register of each sampled HF to assess the screening of children using the Appearance, Pulse, Grimace, Activity, and Respiration (APGAR) score, resulting in 246 records of new-born children. The APGAR score was used as a proxy measure of screening for birth defects, as previous research has established its association with birth defects.^16^

### Data Collection

Data was collected within each HF about the screening of new-born children for birth defects, using the APGAR score with a sample of 6 children from the period of July 2020 to May 2021. Additionally, data was collected about the availability of 5 core equipment necessary for delivering safe motherhood interventions, including delivery beds, delivery sets, Ambu-bags, protective wear for midwives, bulb syringes, and hand washing facilities. District Health Information Software (DHIS2) data was extracted using a checklist on indicators such as 1st and 4th ANC, family planning, skilled birth attendance, and postnatal care attendance for the financial years 2016/2017, 2017/2018, 2018/2019, and 2019/2020.

The community household data was collected using a structured questionnaire for mothers of children aged 0 to 11 months in each interview location. The questionnaire was completed using the Open Data Kit (KIT) on android mobile devices and uploaded to the survey server.

Explanatory in-depth interviews were conducted with 12 midwives and 14 mothers of children aged 0 to 11 months to provide an account of the observed quantitative findings. Five midwives were selected from 4 HFs that performed exceptionally well, while the other 7 were from 4 worst-performing HFs across the 4 districts. Interviews were conducted until saturation point was reached, meaning that no new information emerged from the participants.^17,18^

### Rating coverage of the Child Disability and Birth defects Rehabilitation (CDR)-Safe motherhood services delivery

The assessment of performance was conducted at 3 different levels: the Health Facility (HF), district, and intervention region level as earlier described. At the district level, if a district did not meet the Decision Rule (DR) set for it, it was considered to not reach the 80% target, hence in need of urgent support in the affected indicator. For the overall project intervention, coverage was considered satisfactory if the performance for that indicator was at least 80%.

### Quality Control

Research assistants were trained for 3 days prior to data collection. One day was spent pre-testing the tools at Kajjansi HC IV and its catchment area in Wakiso district. To ensure coherent understanding among mothers/respondents who did not understand English, the tools were translated into the local language (Luganda) used in the study area. The same sampling and data collection approaches were used across all the HFs and their respective catchment areas.

### Ethics

Ethical approval to conduct this study was sought from Mildmay Uganda Research Ethics Committee (MUREC) – approval number REC REF 0603-2020 and by the Uganda National Council of Science and technology (UNCST) – research registration number HS896ES. The research was carried out in accordance with the Helsinki Declaration's principles. Parents were provided with sufficient information about the risks and benefits of their children participating in this study, as well as consent and confidentiality concerns. Respondents were assured of confidentiality during data collection, including assurances that their names would not appear in any publication. Interviews were conducted in privacy where no one would hear the conversation between the data collector and the respondent. In terms of their records, neither the mothers' nor the children's names were written anywhere in the tools. The study report does not contain any information that can lead to identity of a respondent.

## RESULTS

Table 2 displays the attributes of the survey participants. The majority (51.2%) of the participants were aged between 20 to 29 years. Out of the total number of respondents, Mityana district had the highest number of participants, accounting for 31.7% (78/246) of the total. The majority of the participants (54.5%) were married, while 38.2% of the respondents were from households with a size of 4 to 5 members. Most of the respondents (72%) lived within a 0 to 5 km radius from the health facility. About 52.8% of the participants had completed primary education, and 61% of them belonged to the catchment areas of HC III-level health facilities.

**TABLE 2:** Characteristics of the Respondents (Mothers of Children 0-11 Months) (N=516)

Variable		Frequency	Percentage
District	Kassanda	60	24.4
	Luwero	60	24.4
	Mityana	78	31,7
	Mubende	48	19.5
	Total	246	100.0
Participants per HF level	Regional Referral Hospital	6	2.4
	General Hospital	12	4.9
	HC IV	48	19.5
	HCIII	150	61.0
	HCII	30	12.2
	Total	246	100.0
Age category	Less than 20	31	12.6
	20–29	126	51.2
	30 and above	89	36.2
	Total	246	100.0
Marital status	Cohabiting	64	26.0
	Single	48	19.5
	Married	134	54.5
	Total	246	100
Household size	1–3 members	78	31.7
	4-6 members	94	38.2
	7-10 members	71	28.9
	Greater than 10	3	1.2
	Total	246	100.0
Distance from the HF	0-5 km	177	72.0
	6–10	31	12.6
	>10 km	38	15.4
	Total	246	100.0
Education level	Not educated	29	11.8
	Primary	130	52.8
	Secondary	78	31.7
	Post-secondary	9	3.7
	Total	246	100.0

### Population Level Coverage Estimates for Safe Motherhood Indicators

Table 3 displays the estimates of coverage at the population level. The results indicate that 95.9% (95% CI: ± 2.5) of the mothers reviewed attended at least one ANC visit during their last pregnancy, while only 64.2% (95% CI: ±6) made at least 4 visits. Additionally, only 47.2% (95% CI: ±6.2) of the mothers had initiated ANC attendance in the first trimester of pregnancy. About 56.9% (95% CI: ±6.2) of the mothers had an abdominal ultrasound scan during their last pregnancy. Folic Acid uptake and at least two doses of Fansidar for malaria prevention were high, with coverage rates of 91.7% (95% CI: ±3.4) and 94.3% (95% CI: ±2.9) respectively. However, only 79.3% (95% CI: ±5.1) of the mothers were counselled about breast-feeding during pregnancy. The lowest coverage rate was observed for testing for syphilis during pregnancy, with only 35.0% (95% CI: ±6) of the mothers of children aged 0 to 11 months being tested for syphilis during their last pregnancy.

**TABLE 3:** Population-Level Coverage Estimates by Core Safe Motherhood Indicators

Indicator	Mubende (n=48)	Kassanda (n=60)	Mityana (n=78)	Luwero (n=60)	Overall coverage (N=246)
ANC services: # *and % mothers of children aged 0–11 months who;*					
“had at least one ANC check during the last pregnancy	46 (95 8%: 95% Cl±5.7)	58 (96.7%; 95% Cl±4.5)	73 (93.6%; 95% Cl±5.4)	59 (98.3%; 95% Cl±3.3)	236 (95 9%; 95% Cl±2.5)
“ had their first ANC check during the first trimester in the last pregnancy	19 (39.6%; 95% Cl±13.8)	21 (35%; 95% Cl±12.1)	42 (53.8%: 95% Cl±11)	34 (56.7%; 95% Cl±12.5)	116 (47.2%; 95% Cl±6.2)
“ had at least 4 ANC checks during the last pregnancy	34 (70.8%; 95% Cl±11.7)	43 (66 7%; 95% Cl±11.9)	48 (61.5%; 95% Cl±10.8)	33 (55%; 95% Cl±12.6)	153 (64 2%; 95% Cl±6)
“ had an abdominal ultrasound scan during the last pregnancy	21 (43.6%; 95% Cl±14)	26 (43.3%; 95% Cl±12.5)	51 (65.4%; 95% Cl±10.6)	42 (70%; 95% Cl±11.6)	141 (56.9%; 95% Cl±6.2)
“were screened for syphilis during the last pregnancy	19 (31.7%; 95% Cl±11.8)	12 (25%; 95% Cl±12.3)	33 (42.3%; 95% Cl±11)	22 (36.7%; 95% Cl±122)	86 (35.0%; 95% Cl±6)
“were given folic acid during the last pregnancy	43 (89.6%; 95% Cl±8.6)	53 (88.3%; 95% Cl±8.1)	72 (92.3%; 95% Cl±5.9)	58 (96.7%; 95% Cl±4.5)	226 (91 7%; 95% Cl±3.4)
“ received at least 2 doses of Fansidar* during the last pregnancy	47 (97.9%, 95% Cl±4.1)	53 (88 3%; 95% Cl±8.1)	74 (94.9%; 95% Cl±4.9)	58 (96.7%; 95% Cl±4 5)	232 (94 3%; 95% Cl±2.9)
“ were provided counselling about breastfeeding during the last pregnancy	34 (70 8%: 95% Cl±12.9)	46 (76.7%; 95% Cl±10.7)	64 (82.1%; 95% Cl±8.5)	51 (85%; 95% Cl±91	195 (79.3%; 95% Cl±5.1)
Delivery services: # *and % mothers of children aged O-ll months who;*					
“ gave birth at a HF with a skilled health care provider	43 (89.6%; 95% Cl±8–6)	47 (78.3%; 95% Cl±10.4)	66 (84 6%; 95% Cl±8)	59 (98.3%; 95% Cl±3.3)	215 (37.4%; 95% CI ±4.1)
# and % children screened for birth defects at birth**	32 (66.6%; 95% Cl±13.3)	47 (78.3%; 95% Cl±10.4)	76 (97.4%; 95% Cl±3.5)	59 (93.3%; 95% Cl±3.3)	234 (87%; 95% Cl±4.2)
PNC services: *# and K mothers of children aged O-ll months who;*					
“ received a postnatal check within six days of giving birth	28 (58.3%; 95% Cl±13.9)	41 (68.3%; 95% Cl±118)	49 (62.8%; 95% Cl±10.7)	37 (61.7%; 95% Cl±123)	155 (63%; 95% Cl±6)
“ initiated breastfeeding within the 1st hour of birth	30 (62.5%; 95% Cl±13–7)	48 (30%; 95% Cl±10.1)	46 (59%; 95% Cl±109)	33 (55%; 95% Cl±12.6)	157 (63.8%; 95% Cl±6)
“ whose newborns were administered OPVO immediately at birth	39 (83.3%; 95% Cl±10.6)	51 (85%; 95% Cl±9)	71 (91%; 95% Cl±6.4)	50 (83.3%; 95% Cl±9.4)	211 (85.8%; 95% Cl±4.4)
Knowledge of mothers on child disability prevention and management *# and % mothers of children OQed 0–11 months who;*					
“ correctly mentioned at least three causes of child disability	32 (66.7%; 95% Cl±13.3)	38 (63.3%; 95% Cl±12.2)	44 (56.4%; 95% Cl±11)	34 (56.7%; 95% Cl±12.5)	148 (60.2%; 95% Cl±6.1)
“ correctly mentioned at least four ways of preventing child disability and/or birth defects	30 (62.5%; 95% Cl±137)	41 (68.3%; 95% Cl±10.6)	51 (65.4%; 95% Cl±10.6)	31 (51.7%; 95% Cl±12.6)	153 (62.2; 95% Cl±6.1)
“mentioned that they would take their child to a Hf if they suspected that the child had a disability or birth defect	44 (91.7%; 95% Cl±7.8)	56 (93.3%; 95% Cl±6.3)	76 (97.3%; 95% Cl±3.6)	59 (98.3%; 95% Cl±6.3)	235 (95.5%; 95% Cl±2.6)

** Indicator coveraqe computed usinp] data from the rnaternity/delivery repjister

The results further show that 87.4% (95% CI: ±4.1) of women received skilled attendance during birth, and 87% (95% CI ±4.2) of the reviewed children born in the health facilities had their APGAR score recorded in the maternal register to investigate for birth defects. Additionally, 63% (95% CI: ±6) of mothers attended postnatal care within 6 days after giving birth, and 63.8% (95% CI±6) initiated breastfeeding within the first hour after delivery. Finally, the population coverage for children aged 0 to 11 months who received Oral Polio Vaccine (OPV0) immediately after birth was 85.8% (95% CI: ±4.4).

To assess their understanding of the causes of child disability, the mothers were asked to name at least 3 causes of child disability or birth defects. Only 60.2% of the mothers demonstrated knowledge on the causes of child disability. To assess their understanding of how to prevent child disability, the mothers were asked to name at least 4 ways of preventing child disability and birth defects. 62.2% (95% CI ±6.1) of the mothers had knowledge on how to prevent child disability and birth defects. Furthermore, the mothers were asked if they knew the right place (a health facility) where to take their children for examination and investigation if they suspected their child had a disability. Of the 246 mothers interviewed, 95.5% (95% CI ±2.6) knew where to take their child for examination and investigation (i.e., to a HF). Population-level coverage estimates for core safe motherhood indicators are presented in Table 3.

### LQAS Classification and Qualitative Explanation of coverage of the Safe Motherhood Indicators for the Districts Antenatal care (ANC) services utilisation

Table 4 presents the results of the LQAS assessment of the coverage of the core safe motherhood indicators for the study districts. The ANC indicators show that no district achieved the target coverage of 80% based on the corresponding DR; including ANC attendance in the first trimester, ultrasound scanning during pregnancy, and screening for syphilis during pregnancy. Only 37.5% (95% CI: ±14.8) of the health facilities showed optimal use of ultrasound scan services among pregnant women. It was found that most mothers do not see the need to take an ultrasound scan after being physically examined by the midwife during ANC and after being told that their child is fine. This is supported by the statement of one mother who said that; “*I did not go for the scan because I was told everything was okay*”.

**TABLE 4:** LQAS Classification of Health Facilities by Core Safe Motherhood Indicators

Indicator	Mubende (n=8) DR=6	Kassanda (n=10) DR=7	Mityana (n=13) DR=9	Luwero (n=10) DR=6	Coverage	(95% Cl)
ANC services HFs with at least 5 of the sampled 6 mothers of children aged 0-11 months in their catchment area;						
“who had their first ANC check during the first trimester in the last pregnancy	0	0	4	3	17.1%	±11.5
“who had at least one ANC check during the last pregnancy	8*	10*	12*	10*	97.5%	±4.8
“who had at least4 ANC checks during the last pregnancy	5	4	4	5	42.5%	±15.2
“ who had an abdominal ultrasound scan during the last pregnancy	1	2	7	5	37.5%	±14.8
“ who were screened for syphilis during the last pregnancy	0	1	3	1	12.2%	±10
“ who were ever given folic acid during the last pregnancy	5	8*	12*	10*	87.8%	±7.4
“ who received at least *2* doses of Fansidar* during the last pregnancy	7*	8*	13*	10*	95.1%	±6.6
“ who were provided counselling about breastfeeding during pregnancy	4	7*	10*	8*	72.5%	±13.7
Delivery services HFs with at least 5 of the sampled 6 mothers of children aged 0-11 months in their catchment area;						
“who gave birth at a HF with a skilled health care provider	6*	6	10* 10*	78%	±12.7
HFs with children screened for birth defects at birth**	4	9*	12*	10*	85.0%	±10.9
PNC services HFs with at least 5 of the sampled 6 mothers of children aged 0-11 months in their catchment area;						
“ who received a postnatal check within six days of giving birth	5	5	7	7*	60.0%	±15
“ who initiated breastfeeding within the 1st hour of birth	3	7*	4	2	36.4%	±14.7
whose newborns were administered OPVO Immediately at birth	5	8*	12*	7*	78.0%	±12.7
Knowledge of mothers on child disability prevention and management HFs with at least 5 of the sampled 6 mothers of children aged 0-11 months in their catchment area;						
“who correctly mentioned at least three causes of child disability	3	5	7	3	43.9%	±15.2
“who correctly mentioned at least four ways of preventing child disability	5	4	5	4	43.9%	±15.2
“ mentioned that they would take their child to a hf if they suspect a disability in the Child (knowledgeable Of what to do if they suspect a child to have a disability)	6*	9*	13*	10*	92.7%	±8.0
Number of indicators in which the district has acceptable coverage based on DR	5	9	9	10		

* District's number of HFs with acceptable performance has reached the decision rule and therefore, the district attained the 80% perfromance threshold

** Indicator coverage computed using data from the maternity/delivery register

The availability of ultrasound services is lower in HC II facilities, with only 36.7% of respondents reporting access. However, access improves at higher levels of the healthcare system. Mothers living in rural areas where ultrasound services are not available face access challenges and are unlikely to travel even if they have been advised to take an ultrasound scan, as they perceive it as expensive due to involved transportation costs. Additionally, in most communities, the desire to determine the sex of the child is a more common reason for having an abdominal ultrasound scan than the checking for foetal abnormalities or disabilities, as reported by one respondent.

*Most women are trying to find out the sex of the child. They will ask for the money for the scan for that. They come back and tell you that this is a boy but never tell you in case of any disability on the child's finger, foot or eyes. They only tell you the sex of the child* (Midwife, HC III, Mityana district).

The study found that in all study districts, none of the HFs had unacceptable coverage regarding 1^st^ANC attendance; 97.5% (95% CI: ±4.8) of the HFs had at least 5 out of 6 sampled mothers attend ANC at least once. However, only 42.5% (95% CI: ±15.2) of the HFs had optimal performance in terms of ANC utilisation continuity of at least 4 visits. Furthermore, only 17.5% (95% CI: ±11.5) of the HFs had satisfactory performance with regards to mothers initiating their first ANC visit during the first trimester. Additional in-depth interviews with stakeholders revealed that the late commencement of ANC was a key factor contributing to the failure to achieve the minimum required 4 ANC visits.

*We have trained the mothers of this place about goal-oriented antenatal and we have informed them to start antenatal as soon as one realises that she is pregnant. However, some mothers in this place think that if you go early, they will take drugs for all the times they visit* (Midwife, Mubende RRH).

It was also pointed out that some mothers will come late just to get the ANC card;

*They only come to the antenatal clinic because they want to get an antenatal card. Because of this, they will come late in the pregnancy that even some deliver from antenatal* (Midwife, Kasambya HC III).

The study revealed that there is generally low motivation among mothers to use ANC services. Many mothers only attend ANC to receive the (ANC) card that is often asked for at HF during child birth. Additionally, some husbands are hesitant to provide their wives transport funds and other expenses for multiple ANC visits, which they perceive as a financial burden. Some women also believe that making as many as 4 ANC visits is unnecessary, especially if they have not been informed of any pregnancy-related problems.

Screening pregnant mothers for syphilis is vital in identifying those who may have the infection, which, if left untreated, could lead to stillbirth, miscarriage, or harm to the baby. However, the study found that only 12.5% (95% CI: ±10) of the HFs had optimal performance in this regard.

A high proportion, 87.8% (95% CI: ±7.4) of HFs demonstrated optimal performance regarding folic acid supplementation to pregnant women during ANC, and no district fell short of the DR.

Regarding HFs' provision of counselling on breastfeeding during pregnancy, Mubende was the only district that fell short of the DR.

The district-level analysis indicates that Mubende had the poorest performance, failing to reach the coverage target in 6 of the 8 ANC indicators. In contrast, each of the remaining districts failed to attain optimal performance in 4 of the 8 ANC indicators.

### Delivery Services

Less than half of the districts had unsatisfactory coverage for the 2 delivery service indicators that were assessed. Only one district, Kassanda, fell below the 80% coverage target for deliveries at HFs with skilled assistance; 78% (95% CI: ±12.7) of HFs in Kassanda demonstrated optimal performance.

### Postnatal Services

The study found that the administration of Oral Polio Vaccine (OPV) at birth (OPV0) had the highest level of optimal performance among Postnatal Care (PNC) indicators, with 78% (95% CI: ±12.7) of Healthcare Facilities (HFs) meeting the coverage target. In contrast, the initiation of breastfeeding within the first hour after delivery had the lowest coverage of HFs with optimal performance; only 36.4% (95%CI: ±12.7) of the HFs reached the coverage target. Regarding OPV0 provision, Mubende district fell below the 80% coverage target, while all the districts but Kassanda did not reach the coverage target for early initiation of breastfeeding. The overall coverage of HFs with optimal performance in reviewing mothers within 6 hours after delivery was 60% (95% CI: ±15), 7 of the 8 districts (except Luwero) did not attain the 80% coverage target. Furthermore, only 50.0% (95% CI: ±15.3) of HFs had optimal performance in postnatal care attendance at day 6; no district reached the 80% coverage target.

### Knowledge about Child Disability Prevention and Management

The level of maternal knowledge on the causes of child disability was found to be unsatisfactory, with only 43.9% (95% CI: ±15.2) of HFs achieving optimal performance, and no district reached the 80% coverage target. Similarly, the proportion of HFs with optimal performance regarding mothers' knowledge on how to prevent child disability was low at 43.9% (95% CI: ±15.2); all the districts did not reach the 80% coverage target for this indicator except Mubende. However, most mothers reported that they would seek care at a health facility if they suspected their child had a disability, with 92.7% (95% CI: ±8.0) of HFs achieving optimal performance in this regard – no district fell short of the 80% coverage target in this indicator.

In general, Mubende district had the lowest performance, falling short of the 80% coverage target in 13 of the 18 indicators. According to data from the Ministry of Health's Health Management Information Systems, Mubende district had more complex health system challenges, as evidenced by it's poor safe motherhood and maternal and child health performance and was thus prioritised for initial implementation. The same difficulties may have hampered the interventions' effectiveness. Furthermore, the later implementation districts may have benefited from the experience obtained by the project team while dealing with Mubende and accordingly adjusted their implementation approach. Mityana and Kassanda followed, each with 9 indicators short of target. Luwero district had the best overall performance, failing to reach 80% of its HFs with optimal performance in only 8t out of the 18 SMH indicators.

Providing safe motherhood services in lower-level HFs of HC II has been challenging, as these facilities lack the necessary equipment for deliveries. Referring mothers to other facilities for delivery can also be complicated, particularly if they have travelled from far away. As a result, many women end up delivering with the assistance of traditional birth attendants, who are often numerous in communities served by HC IIs. A midwife at a Health Centre II provided some insight on this issue;

*The HF is still a lower-level facility. We lack most of the things because it's not a centre where you can offer most of those services for safe motherhood properly. There are a lot of things that we need and don't have. You find most mothers don't deliver from the HF. They have those Traditional Birth Attendants (TBA) they go to who are very many within the community. The woman will also not come to the facility citing lack of services and that's how they find themselves going to the TBA. They also loose that morale* (Midwife, HC II, Kassanda district)

As a result, some midwives, particularly those in HCII, report that the project has not progressed well as their role is limited to creating awareness about SMH and childhood disability. Furthermore, health workers have been successful in providing safe motherhood talks, particularly through public platforms. As they are aware that they may not receive some SMH services, some pregnant women and caregivers of children with disabilities may choose to bypass lower-level HCs and seek services at hospitals where they expect to receive better care.

*You know, we love the hospital and despise the lower-level HFs. In case I get pregnant I go there because it is now nearby. Previously it was new and I used to despise it. It has been here but like I told you we love the hospital. You see we even travel to Mubende leaving Lusalila here (*Mother of 8 months aged child, Lusalira-Mubende).

This partly explains why the SMH and CDR indicators may not show optimal performance at lower-level HFs if they are located close to hospitals or health centre IVs.

## DISCUSSION

The aim of this study was to determine the coverage of Safe Motherhood (SMH) and early child services that are crucial for preventing birth defects. The results indicate that all districts had achieved satisfactory coverage (≥80%) in terms of healthcare facilities (HFs) with optimal performance in the first Antenatal Care (ANC) visit and administering intermittent preventive treatment of malaria with at least two doses of Fansidar. The HFs in the entire project area had satisfactory performance in providing Folic Acid during pregnancy, but Mubende district fell below the target. Other indicators with satisfactory coverage included screening for birth defects at birth (except for Mubende district), mothers with knowledge of where to take a child suspected of having a disability (all districts achieved the target), and availability of required SMH tools (all districts had acceptable performance). However, in 7 indicators (utilisation of ultrasound scan during pregnancy, first ANC attendance in the first trimester, syphilis screening, postnatal care visit at day 6, early initiation of breastfeeding, mothers knowledgeable about the causes of child disability, and mothers with knowledge of how to prevent childhood disability), no district achieved the 80% coverage target for their HFs' performance.

The findings suggest that while there is a high uptake of the first ANC visit, there is low continuity of care as evidenced by the poor coverage of HFs and districts with optimal performance in mothers who attend at least 4 ANC visits. Late initiation of ANC is a significant factor contributing to poor continuity of care, not only in the project area but also in Uganda as a whole.^19,20^ The drop in continuity of ANC service utilisation can be attributed to various factors including; long distances to health facilities, low functionality of lower-level HFs, and poor perceived quality of care. Mothers often prefer tertiary care from hospitals located far away, which partly explains the low uptake of ANC services. However, the poor performance of Mubende district, which hosts the only regional referral hospital in the project area, challenges this assumption. It is possible that mothers who prefer hospital-based ANC services do not continue with ANC services due to the long distance. This discontinuity affects the demand for other services like ultrasound scans and counselling on breastfeeding, which are usually scheduled later in pregnancy during goal-oriented ANC.^21^

Although the first ANC attendance is high, screening for syphilis is a service that has poor overall coverage. This suggests that the HFs are not providing adequate testing services for syphilis during the first ANC visit.^22,23^ In the future, efforts to improve the screening for syphilis and ultrasound scan services should prioritise empowering the HFs to provide these services before mobilising communities to demand them. The ANC provides an opportunity for prevention, detection, and preparation for the timely management of childhood disability once detected. The low continuity in ANC uptake in the project area implies that there is a missed opportunity for these essential services, including health education on birth defects and disability prevention, micronutrient supplementation such as folic acid to prevent neuro tube defects like spina bifida, iron supplementation to prevent anaemia, and prophylaxis against malaria. Failure to prevent malaria may lead to stillbirths, premature deliveries, or underweight babies.

Initiating ANC early during pregnancy is crucial for screening and managing conditions such as diabetes, which if left untreated, can lead to birth defects of the spine, brain, and limbs, such as sacral agenesis and holoprosencephaly. Delayed initiation of ANC also means missing out on opportunities for early screening of family history related to birth defects and education on preventing birth defects associated with drug intake during the first trimester.^24^ This may also result in missed opportunities for early detection of birth defects, which could lead to delayed corrective measures. When mothers are not screened for syphilis, those who have the disease but remain undetected will miss out on the necessary treatment, which is essential for preventing the complications that the disease can cause.^9,25^ The low coverage in this indicator suggests that screening for other diseases that can cause birth defects, such as sexually transmitted infections, rubella, and non-communicable diseases, may also be missed.

Apart from physical examinations and screening for danger signs during pregnancy, the World Health Organization recommends ultrasound scans to be conducted before the 24th week of pregnancy to detect any foetal anomalies and improve the overall pregnancy experience for women.^26^ One of the project's interventions aimed to increase the use of abdominal ultrasound scans during pregnancy by educating pregnant women about their importance and recommending that they receive at least one scan. However, this study found that both the supply and demand for ultrasound services were low in all project districts. It was unclear whether the scans were being used to detect birth defects, as we did not collect information on the gestational age or purpose of the scan. Some women reported that they found the scans too expensive, while others mentioned that the additional transportation costs required to travel to HFs that provided the service is a burden. These findings are consistent with a recent study on the availability of diagnostic technologies in low and middle-income countries, including Uganda, which found limited access to ultrasound scans in basic and middle-level HFs that constitute the majority of facilities in Uganda's healthcare system.^23^ Furthermore, it has been noted that many mothers only opt for an ultrasound scan to determine the gender of the baby. This highlights the need for increased education and awareness on the importance of ultrasound scans in detecting and preventing birth defects and congenital anomalies.

The coverage of deliveries at healthcare facilities with a skilled provider came close to meeting the 80% target in all districts except Kassanda. Additionally, all districts achieved the target of 80% coverage for newborns examined for birth defects and receiving the OPV0 vaccine at birth. The achievement of 80% coverage for skilled birth attendance is particularly noteworthy, considering the challenges posed by the COVID-19 pandemic. During the pandemic, the national facility delivery rate decreased to 62%, significantly lower than the expected 89%.^19^ It is important to acknowledge that communities residing within the catchment areas of HC IIs still encounter the issue of inadequate skilled birth attendants, resulting in many mothers giving birth with traditional birth attendants. This problem is caused by difficulties in accessing HFs such as HC III and above, which is where skilled birth attendance typically occurs, according to Uganda's healthcare system.

Despite the interventions in all the districts, the low coverage of postnatal care (at 6 days) is a concerning finding. This may have implications on the detection of birth defects and disabilities in babies that may have been missed during birth. Moreover, mothers may miss out on timely education regarding the possible causes of child disabilities in the postnatal/neonatal period, ways to prevent them, and actions to take if they suspect their child to have a disability. Additionally, some children may have been born in environments that put them at risk of infections, physical harm, and injury, which can only be ascertained during postnatal care.^27^

Although all the districts showed satisfactory performance in terms of mothers knowing what to do if they suspect their child has a disability, the lack of knowledge regarding the causes of birth defects and how to prevent them remains a barrier to the prevention of childhood disabilities.^28^ This can hinder parents and caregivers from taking the necessary steps to prevent birth defects and manage disabilities at an early stage. For instance, if a mother is not aware that certain medications taken during pregnancy can cause birth defects, she may unintentionally put her unborn child at risk. Similarly, if parents are not familiar with the early signs of developmental delay, they may not seek timely medical attention, leading to more severe disabilities. Therefore, educating and raising awareness about the causes of birth defects and how to prevent them remains an important aspect to empower parents and caregivers to take proactive steps to prevent birth defects and manage disabilities early, resulting in better health outcomes for children.

## CONCLUSIONS

This study demonstrates that there is a discrepancy in the performance of HFs in the critical SMH indicators across various districts. All districts achieved the target coverage in some indicators, such as ANC first visit, mothers receiving two doses of Fansidar for malaria prevention, mothers having knowledge of what to do in case of child disability, and availability of basic tools for SMH services. Folic acid supplementation during pregnancy and screening for birth defects using the APGAR score exceeded the target coverage overall, but at least one district fell below the coverage target in each indicator. All districts fell below the target coverage in indicators such as the first ANC visit in the first trimester, ANC-4, mothers having at least one ultrasound scan during the last pregnancy, screening for syphilis, PNC check at day 6, mothers having knowledge of the causes of child disability, and mothers knowing how to prevent child disability.

Despite being the first district to implement the initiatives, Mubende had the lowest overall performance of the districts, failing to meet the target in 13 of 18 indicators, most likely because the health system bottlenecks that made it to be prioritised for intervention earlier than the other districts have persisted. Luwero district performed best, falling short of the coverage target in the least (8) number of indicators, while Mityana and Kassanda each failed to reach the target in 9.

To address the differences in performance across districts and indicators, it is recommended that project planners adjust their strategies for indicators or districts that are performing poorly or falling behind in some of the well-performing indicators, taking into account the barriers identified in the study. Intervention strategies for Mubende district particularly need to be changed or adapted because it has had a longer duration of intervention but with lower coverage compared to districts that started interventions more recently. Additionally, the districts that have demonstrated high performance in indicators with mixed results offer an opportunity for learning. For future similar projects, it is recommended to include peer or collaborative learning sessions for cross-district learning, enabling poorly performing districts to learn from top performers and continuously adapt or adopt strategies.

### Study limitation

Due to a lack of baseline data, the study was unable to attribute the findings to the project. Another qualitative publication, on the other hand, will explain and link the project processes, their implementation, and perceptions of their implementation and effectiveness. Future projects of a similar nature should consider using a control-before-and-after design to accurately measure the impact. This method would allow for the quantification of the project's impact by comparing the project group's outcomes to those of a control group before and after the project's implementation. Although, our community-survey component focused on the catchment areas of each sampled HF, good coverage may not necessarily reflect efforts of the HF in reference as we did not investigate where respondents received the services the study assessed. However, low coverage in a HF's catchment depicts the need for more action from the facility, and this is in line with objectives of this study.
